# A case report of huge pancreas mucinous cystic neoplasm during pregnancy: How doctors think

**DOI:** 10.1097/MD.0000000000034820

**Published:** 2023-11-17

**Authors:** Lidan Wang, Ling Zhu

**Affiliations:** a Department of Obstetrics and Gynecology, Hangzhou Ninth People’s Hospital, Hangzhou, China.

**Keywords:** case report, mucinous cystic neoplasm, pancreas, pancreas mucinous cystic neoplasm, pregnancy, sex hormone-sensitive

## Abstract

**Rationale:**

Pancreas mucinous cystic neoplasm (PMCN) is uncommon, and its occurrence during pregnancy is rare. The management of PMCN during pregnancy, including diagnosis and surgical timing, is a great challenge.

**Patient Concerns:**

A nontender epigastric mass of the upper abdomen was detected by palpation in a 35-year-old woman, gravida 2, para 1, during the 36th week of gestation. She was referred to our institution for further evaluation.

**Diagnoses:**

Magnetic resonance imaging (MRI) showed a multilocular cystic mass in the body and tail of the pancreas (16.7/12.1/17.6 cm), well-circumscribed with a hyper signal on T2-weighted MRI images. The diagnosis of a pancreatic cyst, probable mucinous, was established.

**Interventions:**

The patient was informed of the possibilities of malignancy, rapid growth, and rupture of the tumor. After a laparotomy and cesarean section, a large cystic tumor was discovered adherent to the pancreas, spleen, mesocolon, and retroperitoneum. The spleen was preserved since there was no evidence of invasion. According to macroscopic examinations, the tumor measured 18 cm was filled with a dark yellow-brownish mucinous fluid and did not appear to communicate with the pancreatic ducts.

**Outcomes:**

After six months of follow-up, there were no signs of recurrence in the patient.

**Lessons:**

PMCN may need to be surgically resected in cases characterized by malignancy risk during pregnancy. As female sex hormones may influence the behavior of PMCN during pregnancy, surgical timing should be determined based on the stage of pregnancy, malignancy status, and condition of the mother and fetus.

## 1. Introduction

Pancreatic mucinous cystic neoplasms (PMCNs) are uncommon and extremely rare.^[1]^ PMCNs account for 2% of all pancreatic tumors and are mostly found in the body and tail (>90%). It is characterized by ovarian-type stroma under a columnar mucinous epithelium.^[2,3]^ PMCNs are almost exclusively female, being known to be sex hormone-sensitive,^[4]^ but their association with pregnancy is an extremely rare phenomenon. To date, there is no available protocol for managing PMCN in pregnancy, including the timing of pancreatic surgery, gestational follow-up, and fetal delivery mode. We experienced a patient with huge pancreatic MCN during pregnancy. Herein, we introduce the case, together with a literature review of MCN during pregnancy.

## 2. Case report

**Figure 1.**
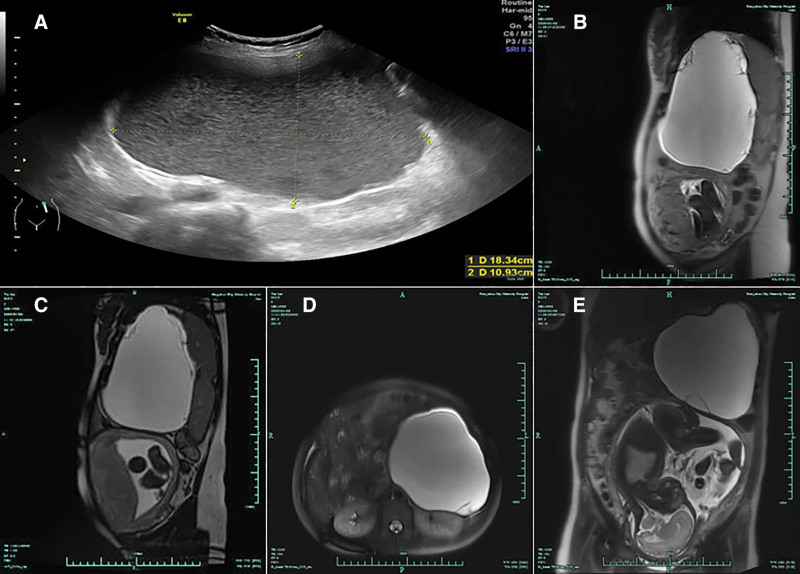
Ultrasound imaging of the PMCN. The tumor, measuring 18.34/10.93 cm, was unilocular, with a thick external wall, with no papillary projection inside (A). Magnetic resonance imaging (MRI) of the patient at 36 weeks of pregnancy. A vertical plan—both the fetus and the PMCN can be seen (B). A transverse plane of the abdomen with the tumor (C). MRI showed a 16.7/12.1/17.6 cm cystic tumor (arrow), in which the contents were hyperintense in T2-weighted imaging (D and E). PMCN = pancreas mucinous cystic neoplasm.

A 35-year-old woman, gravida 2, para 1, presented to our department with a large cystic lesion in the left upper abdomen, and abdominal ultrasound revealed a normal pregnancy. The patient did not have discharge, fever, weight loss, or a history of malignancy in their family. The abdominal ultrasound revealed a cyst in the tail and body of the pancreas measuring 18 cm without a mural nodule (Fig. 1). According to MRI (magnetic resonance imaging), the pancreas body and tail had multilocular cystic masses measuring 16.7/12.1/17.6 cm. The T2-weighted MRI images revealed hyper signal with different signals (Fig. 1). MRI showed there was a possibility of borderline tumors. A pancreatic cyst, likely mucinous, has been diagnosed. Despite its large volume, no subjective complaints were mentioned and no clinical signs were found during abdominal palpation. In this case, there was an elevated level of serum cancer antigen 15-3 (CA 15-3) and squamous cell carcinoma antigen, respectively, at 58.5 U/mL (normal level is < 31.3 U/mL), and 2.0 g/L (normal level is 1.5 < μg/L).

A cesarean section was performed along with resection of the tumor and distal pancreatectomy, due to the tumor’s malignant potential and near-full-term gestation. Because no sign of invasion was observed, the spleen was preserved. The tumor measured 18 cm in diameter, was filled with a dark yellow-brownish mucinous fluid and had no communication with the pancreatic ducts. She gave birth to a healthy girl, 2500 g.

**Figure 2.**
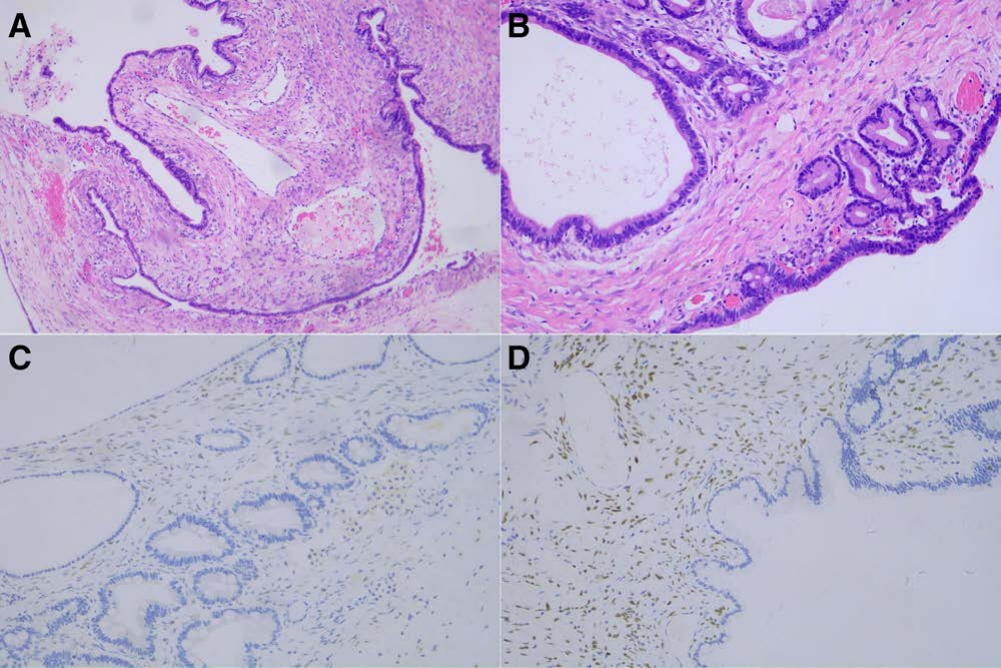
Immunohistochemistry analysis of a pancreatic mucinous cystic adenoma. Mucinous columnar epithelium lined the cyst wall, which was surrounded by ovarian-like stroma (HE, ×100, ×200) (A and B). The nucleus of the ovarian-type stroma was stained partly positively for estrogen receptors by immunohistochemistry (×100) (C). The nucleus of ovarian-type stroma showed partial progesterone receptor staining (×100) (D).

Histology revealed a columnar mucinous epithelium lining the inner walls of the cystic tumor, without atypia or abnormal mitotic activity, with an ovarian-type stroma beneath. A benign PMCN was diagnosed, with tumor-free margins and unaffected lymph nodes (Fig. 2A and B). The immunohistochemistry showed that the receptors for estrogen and/or progesterone were immunopositive (Fig. 2C and D). Additionally, clinical and ultrasound examinations were performed 6 weeks and 6 months after pancreatic surgery and did not reveal any abnormalities. After cyst resection, all blood, serum, and urinary tests were normal.

## 3. Discussion

PMCN and pregnancy are unusually associated. In the English literature, there are 11 reports of patients who delivered vaginally at term,^[1,4–13]^ and in 1 case the tumor was discovered only 2 months after delivery.^[14]^ Of the other 8 PMCNs reported, 6 were resected during the second trimester of pregnancy,^[1,4,6–8,10,11]^ one at 36 weeks due to malignant potential and one at 34 weeks (due to cyst rupture^[15]^). In the majority of these cases, PMCN was discovered incidentally during routine ultrasound examinations during the first 2 trimesters. Due to irregular prenatal visits, PMCN was detected at 36 weeks gestation in our case (Table 1).

**Table 1:** Pancreatic mucinous cystadenoma during pregnancy: reported cases.

First author	Gestational age at the diagnosis (wk)	Tumor diameter (cm)		Complaints	Gestational age at surgery (wk)	Reason for surgery	CA19-9 (U/mL) of cyst fluid	Histology	Pregnancy outcome
		At diagnosis	At surgery						
Herring^[5]^	3	NR	20	NR	17	Malignant potential	22	MCN	VD at term
Ganepola^[9]^	4	5.5	12	Rapid growth	23	Rapid growth malignant potential	NR	MCN (LGMPT) PR(+)	VD at term
Olsen^[12]^	5.5	5	5	NR	18	Malignant potential	NR	MCN	VD at term
Ikuta^[8]^	10	18	18	Distending pain in the left hypochondrial region	10	Malignant potential	34,500	MCN (moderate dysplasia) ER(+), PR(+)	Missed abortion at 10 weeks
Boyd^[7]^	10	19	17	Abdominal distention and fullness An upper abdominal palpable mass	20	High risk of IUGR	NR	MCN (moderate dysplasia)	VD at term
Kato^[11]^	15	2619 ml	4950 ml	NR	23	IUGR	450,000	MCN (benign) ER(+), PR(+)	VD at term
Ishikawa^[6]^	17	12	18	NR	2 months after delivery	Malignant potential abdominal discomfort	>10 000	MCN (benign)	VD at term
Fernandez^[10]^	20	15	15	Episodic epigastric pain	20	Malignant potential	NR	MCN (benign)	VD at term
Tica^[1]^	29	12	15	NR	2 months after delivery	Malignant potential	NR	MCN (benign)	VD at term
Naganuma^[15]^	33	NR	NR	Tumor rupture	34	Tumor rupture		MCN (invasive) PR(+)	CS at 34 weeks
Berindoague^[14]^	2 months after delivery	12	12	NR	3 months after delivery	Malignant potential		MCN (minimal invasive)	VD at term
Hakamada^[13]^	1st trimester, 1st pregnancy	10	10	NR	2nd trimester, 2nd pregnancy	No surgery	NR	NR	VD at term
Kosumi^[4]^	4 months	6	7.6	Left back pai	14th day after delivery	Malignant potential	NR	MCN(benign) ER(+), PR(+)	VD at term
Present case	36	18	18	No subjective complaints	36	Malignant potential	NR	MCN(LGMPT) ER(+), PR(+)	CS at 36 weeks

CS = cesarean section, LGMPT = low-grade malignant potential tumor, MCN = mucinous cystic neoplasm, NR = not reported, PR(+)/ER(+) = immunohistochemical positive staining for progesteron receptors/estrogen receptors/α, VD = vaginal delivery.

To treat PMCN associated with pregnancy effectively, the first and most important step is to schedule pancreatic surgery at the right time, since all PMCNs are considered potentially malignant, an unresected tumor can lead to invasive pancreatic cancer, a potentially fatal condition. Consequently, an accurate diagnosis is crucial, but separating mucinous cysts from nonmucinous cysts, as well as quantifying their malignant potential, are very difficult tasks because all imaging techniques, including MRI, have been inconsistent.^[16,17]^ The accuracy of MRI is estimated to range from 20% to 80%.^[18]^

A study reported that endoscopic ultrasound (EUS) can diagnose pancreatic cysts better than MRI (with a reported accuracy of 40%–93%^[19]^), however, all endoscopies should be avoided during pregnancy. Fine-needle transabdominal aspiration is controversial due to the risk of the seeding of tumor cells.^[6]^ Although theoretically, fine-needle aspiration can be performed, cytology, tumor markers (CEA, CA 19-9, CA 15-3, CA 72-4, CA 125), enzymes (amylase, lipase) as well as DNA analysis from the cyst fluid, have a specificity which varies considerably in different studies.^[20,21]^ As an example, Brugge et al^[22]^ determined that carcinoembryonic antigen (CEA) and CA 72-4 were more accurate than CA 19-9, CA 125, CA 15-3, and cytology and that no combination of tests could be more accurate than CEA alone. Anyhow, if resection of the tumor is mandatory, fine-needle aspiration is not recommended.

Moreover, the serum levels of tumor markers are even less useful for diagnosing PMCN.^[23]^ Bassi et al^[23]^ found that CEA was the best indicator of mucinous cysts, but its sensitivity was only 17%. Its sensitivity can increase to 27% by simultaneously measuring CA 19-9 and CA 125, but only with limited utility in predicting malignancy. However, Sun et al^[24]^ CA19-9 has a moderate ability, and CEA, CA125, and CA724 have a low ability to predict MCNs.

In conclusion, MRI remains the best way to diagnose PMCNs associated with pregnancy, despite its insufficient accuracy.

In general, high-grade malignant potential tumors (HGMPT) have a large size (more than 15 cm), multilocularity, solid masses or papillary projections inside, thick walls and septa, and rapid growth.^[1,6,11]^

As a general rule, all HGMPTs found during the first 2 trimesters of pregnancy should be resected during the second trimester, since there is enough time for the problem to resolve spontaneously before term, with less risk of abortion, and surgery is easier.^[6,8]^

HGMPT diagnosed in the third trimester should be delivered by vaginal delivery followed by surgery.^[7]^ If the tumor shows no or few signs of malignancy (low-grade malignant potential tumor): a single or plurilocular cyst, well-circumscribed, and asymptomatic, the surgery should be postponed until 2 to 3 months after delivery, independent of the moment of its diagnosis during pregnancy.

The second step in managing PMCN during pregnancy is to monitor the pregnancy carefully (if the decision is to simply survey the tumor). The following up of this type of tumor must be done with great attention, due to the malignant potential of all PMCNs and due to possible rapid tumor growth with the occurrence of symptoms,^[1,4–6,8–10,12,14]^ IUGR^[7,11]^ or cyst rupture.^[15]^ PMCNs are almost exclusive to women and may be responsive to female sex hormones.^[4]^ Estrogen receptors and progesterone receptors are expressed in pancreatic MCN.^[25]^ Even though PMCNs usually grow slowly and remain “silent” for many years, high levels of sex hormones during pregnancy are believed to prompt rapid tumor growth. Kato et al^[11]^ reported a pregnant woman’s PMCN, which increased in size from 2619 to 4950 mL in only 46 days, and Ganepola et al^[9]^ reported a case in which a 5.5 cm cyst grew to more than 12 cm within 4 months.

## 4. Conclusions

PMCN may need to be surgically resected in cases characterized by malignancy risk during pregnancy. As female sex hormones may influence the behavior of PMCN during pregnancy, surgical timing should be determined based on the stage of pregnancy, malignancy status, and condition of the mother and fetus.

## Author contributions

**Formal analysis:** Lidang Wang.

**Funding acquisition:** Ling Zhu.

**Investigation:** Lidang Wang.

**Methodology:** Lidang Wang.

**Project administration:** Ling Zhu.

**Supervision:** Ling Zhu.

**Writing – original draft:** Lidang Wang.

**Writing – review & editing:** Lidang Wang.
